# A *de novo* substitution in BCL11B leads to loss of interaction with transcriptional complexes and craniosynostosis

**DOI:** 10.1093/hmg/ddz072

**Published:** 2019-04-03

**Authors:** Jacqueline A C Goos, Walter K Vogel, Hana Mlcochova, Christopher J Millard, Elahe Esfandiari, Wisam H Selman, Eduardo Calpena, Nils Koelling, Evan L Carpenter, Sigrid M A Swagemakers, Peter J van der Spek, Theresa M Filtz, John W R Schwabe, Urszula T Iwaniec, Irene M J Mathijssen, Mark Leid, Stephen R F Twigg

**Affiliations:** 1 Departments of Plastic and Reconstructive Surgery and Hand Surgery; 2 Bioinformatics, Erasmus MC, University Medical Center Rotterdam, CA Rotterdam, The Netherlands; 3 Department of Pharmaceutical Sciences, College of Pharmacy, Oregon State University, Corvallis, OR, USA; 4 Clinical Genetics Group, MRC Weatherall Institute of Molecular Medicine, University of Oxford, John Radcliffe Hospital, Oxford, UK; 5 Leicester Institute for Structural and Chemical Biology, Department of Molecular and Cell Biology, University of Leicester, Leicester, UK; 6 College of Veterinary Medicine, University of Al-Qadisiyah, Al Diwaniyah, Iraq; 7 Department of Pathology, Erasmus MC, University Medical Center Rotterdam, CA Rotterdam, The Netherlands; 8 Skeletal Biology Laboratory, School of Biological and Population Health Sciences, Oregon State University, Corvallis, OR, USA; 9 Department of Integrative Biosciences, Oregon Health & Science University, Portland, OR, USA

## Abstract

Craniosynostosis, the premature ossification of cranial sutures, is a developmental disorder of the skull vault, occurring in approximately 1 in 2250 births. The causes are heterogeneous, with a monogenic basis identified in ~25% of patients. Using whole-genome sequencing, we identified a novel, *de novo* variant in *BCL11B*, c.7C>A, encoding an R3S substitution (p.R3S), in a male patient with coronal suture synostosis. BCL11B is a transcription factor that interacts directly with the nucleosome remodelling and deacetylation complex (NuRD) and polycomb-related complex 2 (PRC2) through the invariant proteins RBBP4 and RBBP7. The p.R3S substitution occurs within a conserved amino-terminal motif (RRKQxxP) of BCL11B and reduces interaction with both transcriptional complexes. Equilibrium binding studies and molecular dynamics simulations show that the p.R3S substitution disrupts ionic coordination between BCL11B and the RBBP4–MTA1 complex, a subassembly of the NuRD complex, and increases the conformational flexibility of Arg-4, Lys-5 and Gln-6 of BCL11B. These alterations collectively reduce the affinity of BCL11B p.R3S for the RBBP4–MTA1 complex by nearly an order of magnitude. We generated a mouse model of the BCL11B p.R3S substitution using a CRISPR-Cas9-based approach, and we report herein that these mice exhibit craniosynostosis of the coronal suture, as well as other cranial sutures. This finding provides strong evidence that the BCL11B p.R3S substitution is causally associated with craniosynostosis and confirms an important role for BCL11B in the maintenance of cranial suture patency.

## Introduction

The flat bones of the mammalian skull and face develop by direct intramembranous ossification of mesenchyme, which is principally derived from mesoderm and neural crest cells (NCCs) (1). Craniofacial sutures are fibrocellular structures that form at the margins of developing bones and limit skull deformation due to both tensile and compressive forces (2). Development and maintenance of sutures is essential and precisely controlled to match skull expansion with the rapid increase in brain size of infants (3).

The major sutures of the skull vault include the metopic and sagittal sutures that separate the paired frontal and parietal bones, respectively, the coronal sutures that form between the parietal and frontal bones and the lambdoid sutures between the parietal and occipital bones. Suture formation, maintenance and subsequent ossification, the latter of which typically begins in the third decade of human life, require the exquisitely choreographed balance of proliferation and differentiation of sutural stem cells and their progeny including bone-forming cells localized at the boundaries of sutures (4).

Craniosynostosis is the most clinically important developmental disorder of the skull vault. Associations of this disorder include restricted skull expansion, increased intracranial pressure and craniofacial dysmorphologies, all of which negatively impact respiration, sensory systems and cognition (5). Failure of the mechanisms that establish the suture or that are responsible for maintaining sutural patency result in craniosynostosis (2).

To date a genetic cause has been identified in approximately a quarter of craniosynostosis cases with important implications for counselling and management (6). Mutations have been detected predominantly in the fibroblast growth factor receptors (*FGFR2* and *FGFR3*), *TWIST1* and *EFNB1* (7,8). More recently, the use of high-throughput sequencing has implicated mutations in many other genes in the aetiology of craniosynostosis, including *TCF12* (9), *SMAD6* (10), *ERF* (11), *CDC45* (12) and *ZIC1* (13). These findings indicate that the causes of craniosynostosis are heterogeneous and involve many different pathways. In addition, craniosynostosis can be present as a consistent, although rare, feature where mutations have a non-specific impact on osteogenesis, for example affecting osteoblast to osteoclast balance or chromatin modification (8).

Heterozygous mutations in *BCL11B* have been linked to human pathologies affecting the immune system, intellectual impairment, developmental abnormalities, as well as dermal and craniofacial defects (14,15). BCL11B plays an essential role in the development of the immune (14,16–22), central nervous (23–25) and cutaneous (26) systems and teeth (27,28). Disruption of a single *Bcl11b* allele in mice results in synostoses within the facial skeleton (29). *Bcl11b^−/−^* mice, as well as mice lacking BCL11B in neural crest-derived cells (*Bcl11b^ncc−/−^)*, exhibit abnormally small and misshapen heads, severe midface hypoplasia and malocclusion. Fusion of multiple facial sutures is seen in these mice, including the internasal, naso-premaxillary, interfrontal, premaxillary-maxillary and temporal sutures. Some *Bcl11b^−/−^* skulls are characterized by a high degree of porosity, mirroring observations in human patients with increased intracranial pressure (29).

*Bcl11b^−/−^*, but not *Bcl11b^ncc−/−^*, mice exhibit severe coronal suture craniosynostosis, which initiates around embryonic day (E) 16.5—E18.5 (29). BCL11B is expressed in the coronal suture beginning at E14.5 and loss of BCL11B results in ectopic expression of both *Runx2* and *Fgfr2c* within the sutural mesenchyme at E16.5, together with concomitant downregulation of *Twist1* expression and ossification of the suture (29). These findings collectively suggest a role for BCL11B in maintaining sutural patency in the mammalian craniofacial skeleton.

Here we describe identification of a patient with craniosynostosis carrying a *de novo* point mutation in *BCL11B* and report the generation of a mouse model that recapitulates the human phenotype. The mutation results in a single amino acid substitution, p.R3S, which severely compromises interaction of BCL11B with transcriptional regulatory complexes containing RBBP4 and RBBP7, but not other transcriptional complexes with which BCL11B interacts. Our data demonstrate that the BCL11B p.R3S substitution underlies craniosynostosis in this patient and that interaction of BCL11B with transcriptional regulatory complexes containing RBBP4/7 plays a key role in the ability of BCL11B to suppress the gene expression program underpinning osteogenic differentiation within mammalian cranial sutures.

## Results

### Clinical description

The index patient was a Caucasian male, delivered by vacuum extraction at 39 weeks of gestation weighing 3390 g. He was the first child of non-consanguineous, unaffected parents. The family history was negative for craniosynostosis. Examined directly after birth, he was noted to have flattening of the forehead and supra-orbital rim on the right side.

**Figure 1:**
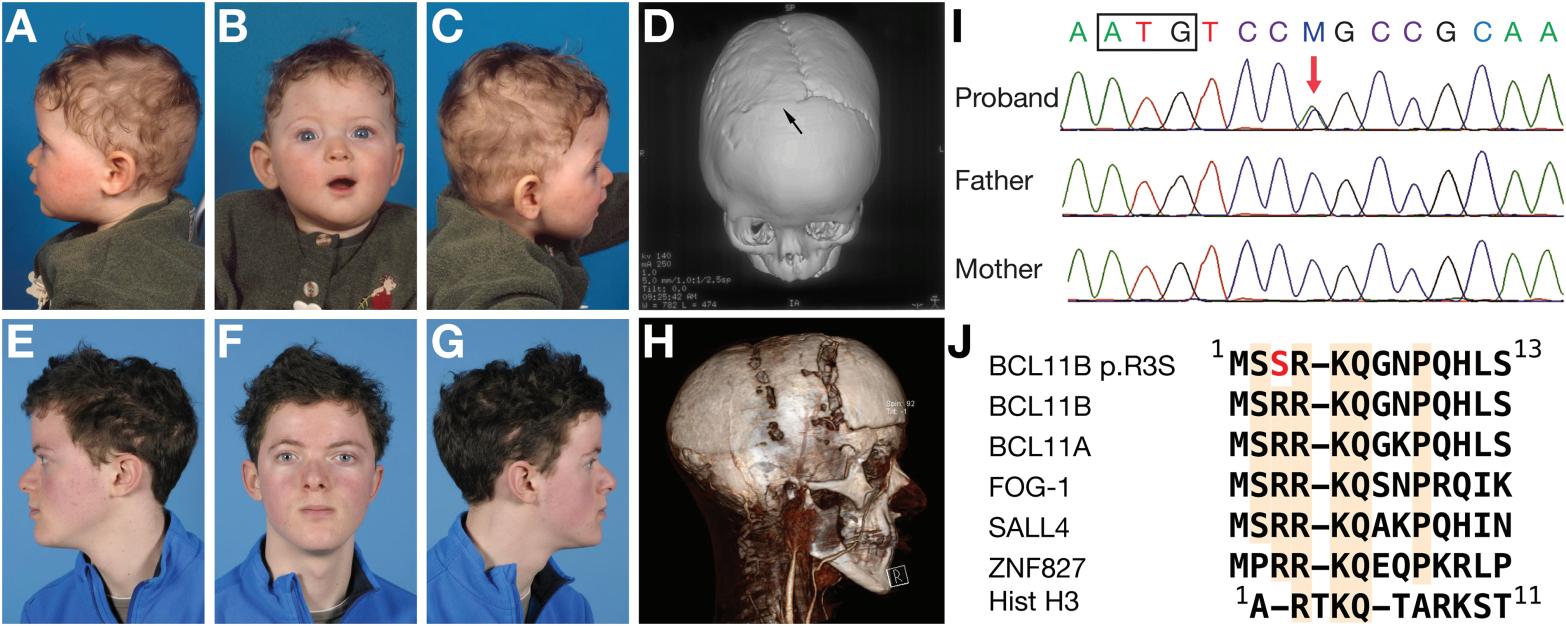
Clinical features. (**A–C**) Pre-operative facial photographs and 3D-CT scan (**D**) at the age of 10 months. Right coronal synostosis is indicated by the arrow. (**E–G**) Post-operative facial photographs at the age of 15 years. (**H**) 3D-CT and angiogram scan at the age of 12 years just prior to distraction. (**I**) DNA sequence analysis of proband and parents showing a *de novo* C>A variant (*red arrow*) in *BCL11B* (c.7C>A). The box encloses the start codon. (**J**) Alignment of amino termini of BCL11B, transcription factors harbouring related sequences and histone H3.

Physical examination at 10 months of age revealed a frontal plagiocephalic head shape, mild vertical orbital dystopia, a high philtrum, a short nose and narrow eyebrows (Fig. 1A–C). The skull circumference was 44 cm (−1.81 SD). Skull radiographs and three-dimensional computed tomography (3D-CT) showed synostosis of the right coronal suture and increased fingerprinting (Fig. 1D). Skull radiographs 2 months later showed partial synostosis of the left coronal suture (not shown). A fronto-supraorbital remodelling was performed at the age of 1 year.

At the age of 5 years, the patient developed impaired vision. Fundoscopy showed papilledema (1.5 dpt right eye and 2 dpt left eye), and a CT scan showed reduced peripheral cerebrospinal fluid spaces, consistent with increased intracranial pressure. This was confirmed by direct measurement (20 mm Hg), and a second fronto-supraorbital advancement was performed. A nonverbal IQ exam, completed at the age of 6 years, indicated a score of 107 (Snijders-Oomen Nonverbal Intelligence Test 2.5-7).

At the age of 11 years, an epileptic insult was observed. Shortly after, vision again declined and fundoscopy showed bilateral papilledema of 2 dpt, while intracranial pressure measurements and imaging (3D-CT and magnetic resonance imaging) were inconclusive. A fronto-orbital advancement and occipital distraction were performed at the age of 12 years. Distraction started after 2 days but was paused after 9 days due to severe headaches. A CT angiogram with 3D-CT reconstruction showed traction on the sagittal sinus and formation of collaterals suboccipitally (Fig. 1H). Distraction was restarted resulting in improvement in vision. Distractors were removed after a period of 4 months. Subsequently, the patient developed normally (Fig. 1E–G) and attended a normal school without additional help up to the age of 16 years.

### Genetic analysis

Karyotyping was normal and *FGFR1*, *FGFR2*, *FGFR3*, *TWIST1* and *ERF* were negative for mutations by dideoxy-sequencing. Whole-genome sequencing (WGS) (30) of the parent–child trio was performed, and the data were analysed following a *de novo* disease model, as described previously (31). Two high-confidence *de novo* coding variants were identified (Supplementary Material, Table S1); the top ranked of which was in *BCL11B* (c.7C>A, encoding p.R3S). Sequence analysis confirmed that this was a *de novo* variant (Fig. 1I). Furthermore, the c.7C>A variant was not present in any database, including the Exome Aggregation Consortium (ExAC) and the Genome Aggregation Database (gnomAD) covering more than 120 000 exomes or genomes from unrelated individuals. The *BCL11B* variant was thought to likely be causally significant, as *Bcl11b* is expressed in cranial sutures (29,32), and *Bcl11b^−/−^* mice exhibit craniofacial abnormalities, including craniosynostosis (29). Furthermore, the p.R3S substitution occurs within a conserved amino-terminal domain found within a class of NuRD complex interacting transcription factors (Fig. 1J). The second-ranked variant was a *de novo* missense change in *FERMT2*. It was considered unlikely to provide a major contribution to the phenotype (Supplementary Material, Table S1). All other coding variants had a low minimal somatic score, and therefore were unlikely to be real. No variants of interest were detected under a recessive or X-linked inheritance hypothesis (data not shown).

To assess the contribution of *BCL11B* variants to craniosynostosis aetiology more generally, we interrogated existing exome sequencing and WGS data (186 cases) and screened by targeted resequencing a panel of 382 unrelated probands, all without a genetic diagnosis. In addition, we analysed *BCL11B* exon 1, which encodes the conserved amino-terminal region of the protein, by dideoxy sequencing in a further 115 cases that were not included in the resequencing cohort. Although we identified several rare variants by resequencing (Supplementary Material, Table S2), no plausible causative changes were identified, indicating that mutations of *BCL11B* leading to craniosynostosis may be rare and/or highly specific.

### Effect of p.R3S substitution on interaction of BCL11B with transcriptional regulatory complexes

**Figure 2:**
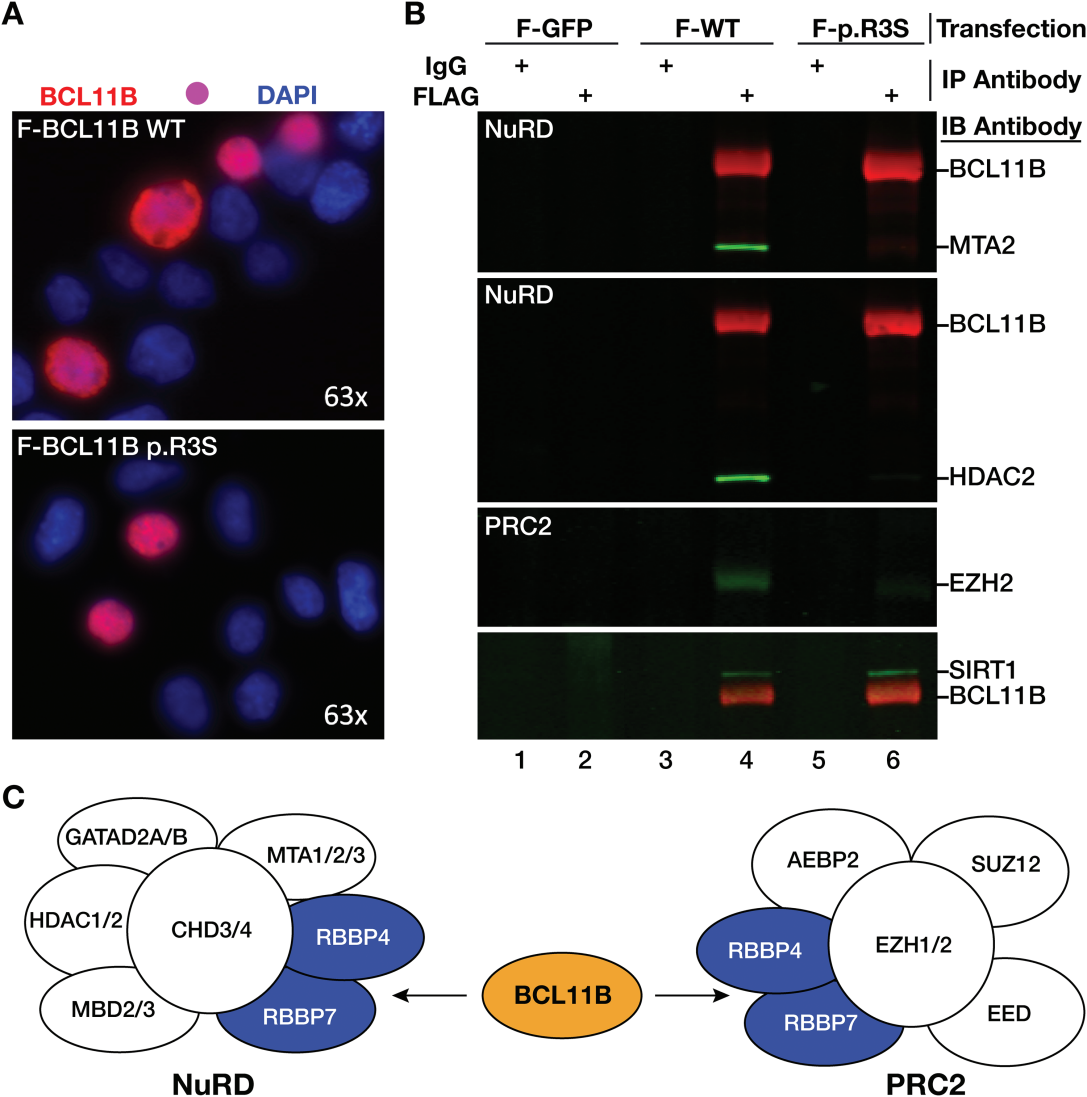
BCL11B p.R3S substitution disrupts interaction with NuRD and PRC2 complexes. (**A**) Immunocytochemistry of HEK293T cells expressing wild-type BCL11B (top) and BCL11B p.R3S (bottom). Cells were stained with an anti-BCL11B monoclonal antibody and co-stained with DAPI to visualize nuclei. (**B**) Co-immunoprecipitation studies. BCL11B and BCL11B p.R3S were immunoprecipitated with the anti-FLAG antibody from transiently transfected HEK293T cells, and the corresponding blot was probed with antibodies to BCL11B (red), MTA2 (green), HDAC2 (green), EZH2 (green) and SIRT1 (green). Comparison of lanes 4 (wild-type protein) and 6 (p.R3S) demonstrates that the patient-derived BCL11B mutant does not interact with the NuRD or PRC2 complexes, however, the p.R3S substitution did not alter interaction of the protein with the class III histone deacetylase SIRT1 (33). (**C**) Schematic outlining BCL11B interaction with the NuRD and PRC2 complexes.

Co-immunoprecipitation (co-IP) analyses were performed in extracts of transiently transfected HEK293T cells to determine if the p.R3S substitution altered the spectrum of selected transcriptional regulatory complexes with which BCL11B interacts. The mutant protein was well expressed and localized in the nucleus in a manner that was indistinguishable from wild-type protein (Fig. 2A). However, co-IP studies revealed that the amino acid substitution abolished the ability of BCL11B to interact with both the NuRD (compare lanes 4 and 6 of top panel of Fig. 2B; MTA2 and HDAC2 were used as markers of the NuRD complex) and PRC2 complexes (compare lanes 4 and 6 of the middle panel of Fig. 2B; enhancer of zeste 2 [EZH2] was used as a marker of PRC2). The patient-derived substitution did not affect the ability of BCL11B to interact with SIRT1, a class III histone deacetylase (compare lanes 4 and 6 of bottom panel of Fig. 2B), with which BCL11B interacts directly (33). Interaction of BCL11B with PRC2, which to our knowledge has not been reported previously, was hypothesized due to the presence of RBBP4 and RBBP7 in the PRC2 complex and is demonstrated herein.

### Interaction of BCL11B and BCL11B p.R3S with the RBBP4–MTA1 complex

**Figure 3:**
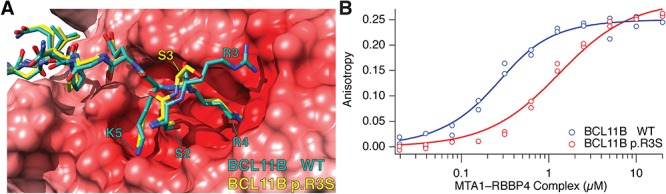
Effect of the p.R3S substitution on interaction of BCL11B with RBBP4–MTA1. (**A**) Modelled wild-type (green) and p.R3S mutant (yellow) BCL11B peptides (residues 1–15) superimposed and bound to the electrostatic potential surface of the RBBP4–MTA1 complex is shown. The electrostatic potential was calculated with APBS (69) and shown in shades of blue and red to represent positive and negative potential, respectively, and visualized with UCSF Chimera (70). (**B**) Binding of fluorescein-labelled wild-type and BCL11B p.R3S amino-terminal peptides to RBBP4–MTA1 complex as monitored by fluorescence anisotropy. Steady-state fluorescence anisotropy measurements of each peptide (12.5 nm) are shown with increasing concentration of unlabelled RBBP4–MTA1 complex. Fluorescence anisotropy was calculated using Eq. 1 and fit to Eq. 2. The dissociation constants for wild-type and BCL11B p.R3S peptides were 0.133 ± 0.043 μm and 1.16 ± 0.28 μm, respectively. Data are from replicate independent experiments, and the error estimate is the 95% confidence interval.

The amino terminus of the NuRD-associated transcription factor FOG-1 shares a conserved sequence with BCL11B (RRKQxxP, see Fig. 1J). We used the high-resolution structure of FOG-1 peptide bound to RBBP4 (34) to construct a model of BCL11B and BCL11B p.R3S peptides bound to the RBBP4–MTA1 complex (35). The resulting model predicts that BCL11B binds to an electronegative surface on one face of RBBP4, principally via three invariant electropositive residues within the conserved, amino-terminal region of BCL11B. Of these, Arg-4 is bound within an electronegative channel of RBBP4, while Arg-3 and Lys-5 interact with clefts closer to the top surface of RBBP4 (Fig. 3A). Recent reports of the crystal structure of BCL11A and SALL4 in complex with RBBP4 described similar findings, as expected (36,37). The p.R3S substitution removes one of these charged RBBP4-coordinated residues and generates a novel potential site for post-translational modification (PTM; e.g. phosphorylation or *O*-glycosylation). To evaluate the consequences of this substitution we used fluorescence anisotropy to measure the equilibrium binding affinity of amino-terminal BCL11B peptides to RBBP4–MTA1 complex *in vitro*. We also conducted molecular dynamics (MD) simulations to gain further insight into this molecular association.

Using fluorescein-conjugated wild-type and BCL11B p.R3S peptides, we estimated equilibrium binding affinities from the increase in anisotropy when titrated with unlabelled RBBP4–MTA1 complex. At physiological ionic strength the p.R3S substitution decreased the affinity of the BCL11B amino-terminal peptide by 8.7-fold, from 0.133 ± 0.043 μM, found with the wild-type Arg-3 peptide, to 1.16 ± 0.28 μM for the mutant, Ser-3-containing peptide (Fig. 3B).

**Figure 4:**
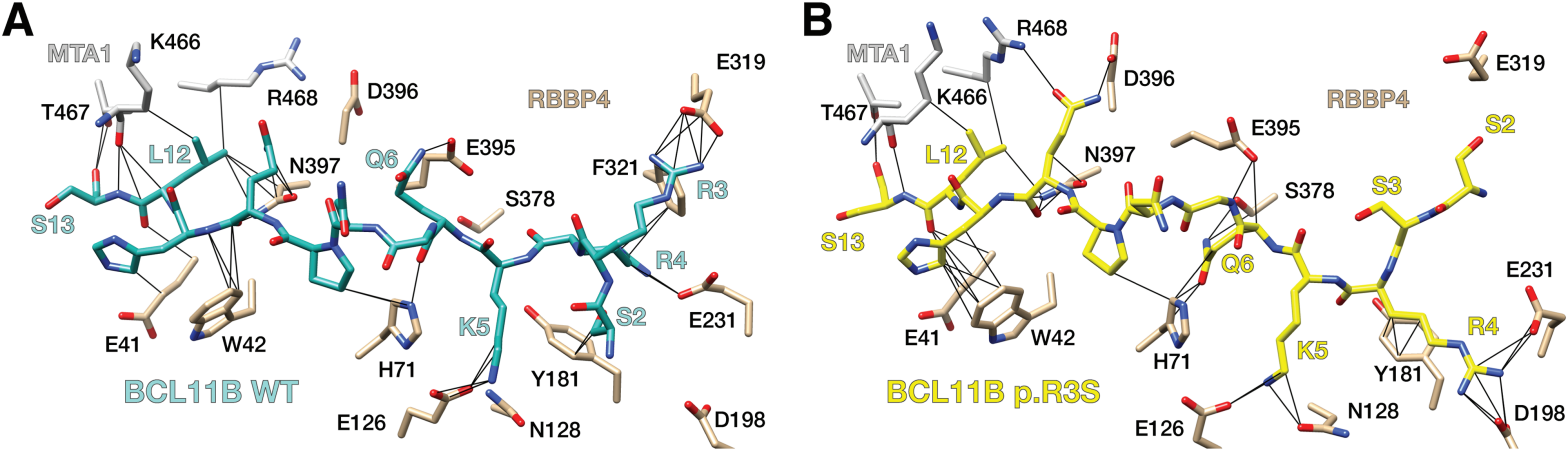
Effect of BCL11B p.R3S substitution on complex bound conformation. RBBP4–MTA1 complex bound structure BCL11B structure of wild-type (**A**) and p.R3S mutant (B) showing the conformation of representative equilibrium structures from MD simulations.

**Figure 5:**
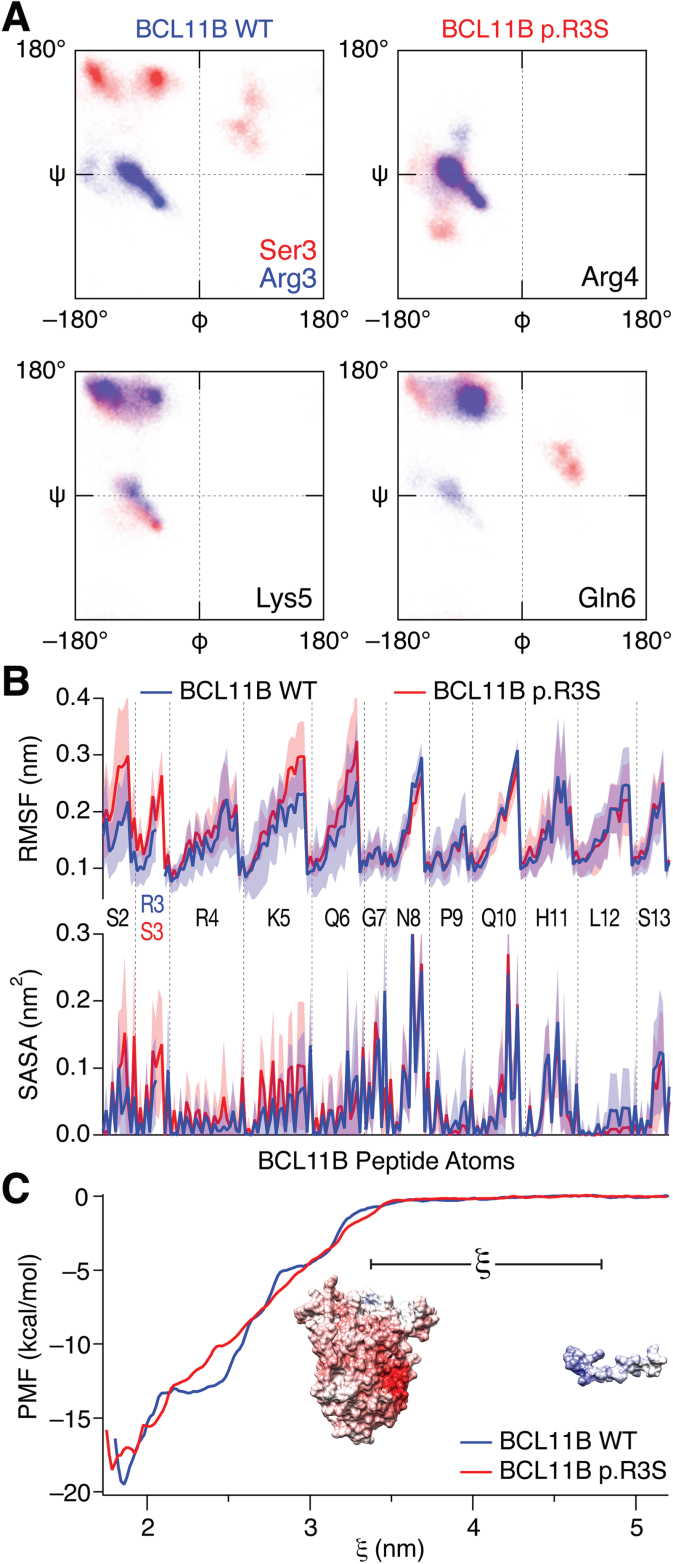
MD analyses of the p.R3S substitution on interaction of BCL11B with RBBP4–MTA1. (**A**) Ramachandran plots of the p.R3S substitution showing the effects on the site of mutation and adjacent residues. The plots show the distribution of backbone configurations from five independent simulations of wild-type (blue) and p.R3S mutant (red) BCL11B peptides bound to RBBP4–MTA1 complex calculated from MD simulations between 50 and 100 ns. (**B)** Root-mean-square fluctuation and solvent accessible surface area of the wild-type (blue) and p.R3S mutant (red) BCL11B peptide from MD simulations. The mean of five independent simulations calculated between 50 and 100 ns is shown, and the shaded regions represent the standard deviations. For wild-type Arg-3, only the backbone atoms in common with mutant Ser-3 are shown. (**C**) PMF from umbrella sampling molecular dynamic simulations. Umbrella sampling simulations for wild-type and BCL11B p.R3S amino-terminal peptides binding to RBBP4–MTA1 complex as a function of reaction coordinate (ξ), which is the centre-of-mass distance between the complex and peptide (illustrated in the inset). The plotted values are the mean of 500 bootstrapped simulations from which the ∆*G*_bind_ was −19.5 kcal/mol for the BCL11B peptide and −18.5 kcal/mol for BCL11B p.R3S peptide.

We conducted MD simulations to assess the consequence of the p.R3S substitution on potential conformational changes in the amino-terminal tail of BCL11B. The resulting equilibrium structures of wild-type BCL11B peptide (Fig. 4A) were similar to the FOG-1-based model (Fig. 3A) and the BCL11A–RBBP4 structure (36), while the BCL11B p.R3S equilibrium structures revealed a conformational change in the peptide backbone at the site of mutation affecting the coordination of nearby residues (Fig. 4B). The mutant Ser-3 side chain was uncoordinated to RBBP4 and its ψ-bond rotational change oriented both itself and Ser-2 away from the RBBP4 surface. The mutant backbone conformation was indistinguishable from wild type at nearby invariant residues Arg-4, Lys-5 and Gln-6 (Figs 4B and 5A), however, the side chains of these residues favoured alternative positions. While the wild-type BCL11B peptide Arg-4 preferred the axial conformation, deep in the RBBP4 binding site as observed in other amino-terminal motif–RBBP4 structures (34,36–38), the p.R3S mutant peptide preferred an RBBP4 surface conformation coordinated to Asp-198. In the wild-type peptide, Lys-5 was observed in multiple conformations, both coordinated in a binding pocket on the RBBP4 surface and uncoordinated and solvent exposed; but in the p.R3S mutant, the uncoordinated solvent exposed conformations were more frequently observed than with the wild-type peptide (Fig. 5B). The side chain of Gln-6 was also more conformationally flexible in the mutant where it assumed a series of Ser-378-localized and Ser-378-coordinated conformations unique to the p.R3S mutant peptide. Simulation results suggest that the p.R3S substitution induces a peptide backbone conformational change that affects adjacent residues. In one direction, this conformational change exposes to solvent both the known PTM site Ser-2 (39) and the mutant Ser-3. In the other direction, the loss of one of the three conserved ionic interactions affects the coordination of Arg-4 and most prominently Lys-5.

We examined the consequence of the BCL11B p.R3S substitution on the statistical binding free energy by calculating the potential of mean force (PMF) from a series of overlapping 15 ns MD simulations taken along the association–dissociation reaction coordinate of the peptide–complex. The analyses calculated a ∆∆*G* of the mutation of 1 kcal/mol (Fig. 5C) a value in agreement with fluorescence anisotropy results (Fig. 3B), adding confidence to the MD-based analyses above.

### Craniosynostosis in mice harbouring the BCL11B p.R3S substitution

**Figure 6:**
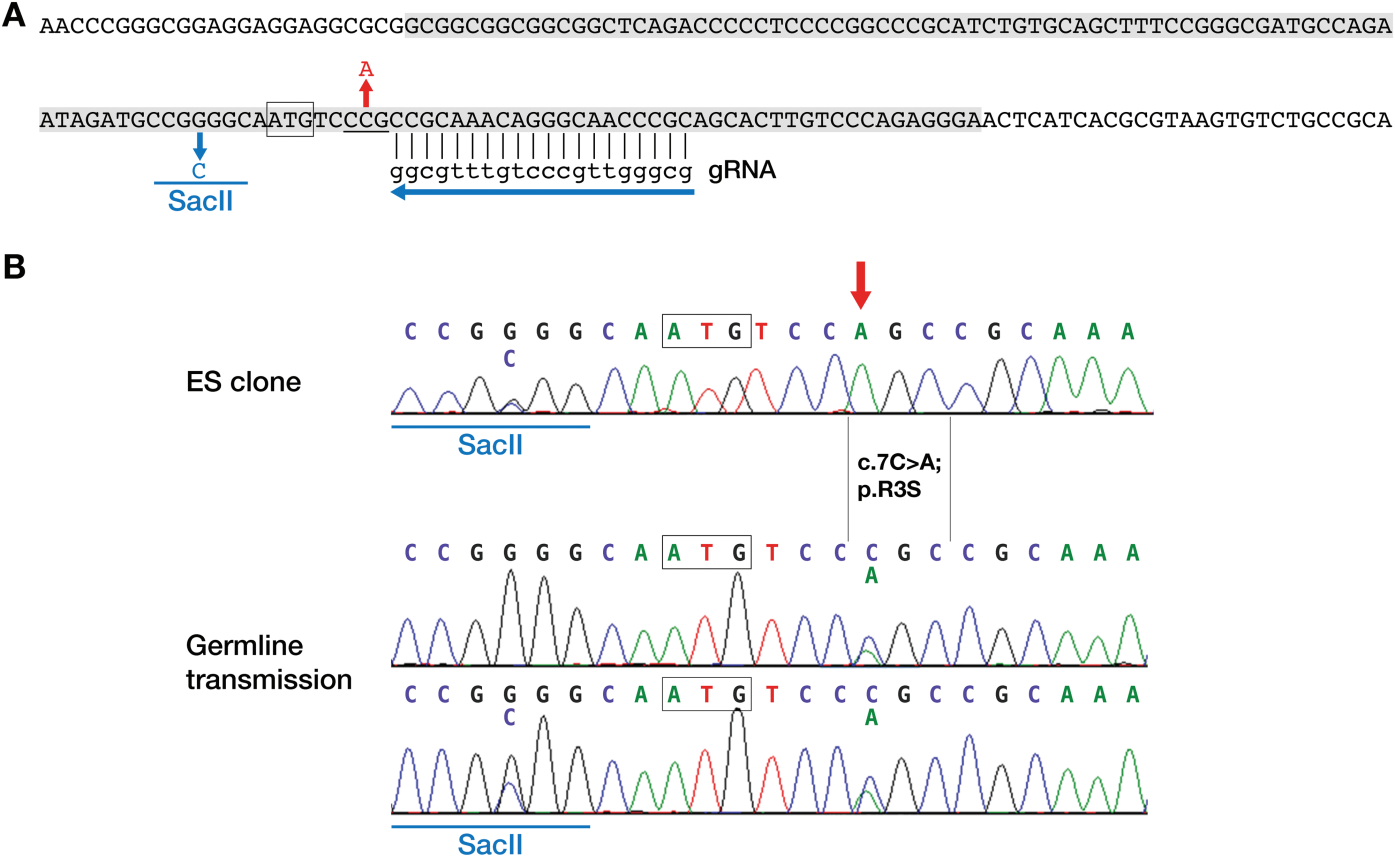
Strategy for generating *Bcl11b*^R3S^ mice. (**A**) Mouse *Bcl11b* genomic sequence around the translation start site (boxed) with CRISPR-Cas9 guide RNA (gRNA) target site and protospacer adjacent motif (underlined) shown. The 127 nucleotides sequence used in the donor oligonucleotide for homology directed repair is shaded. The donor sequence included two mismatches to the genomic sequence to introduce a SacII site for screening purposes (shown in blue) and the c.7C>A; p.R3S mutation (shown in red). (**B**) DNA sequence from an ES clone with a heterozygous SacII site and homozygous c.7C>A mutation (red arrow, top). Germline transmission of c.7C>A with and without the SacII site (below). The translation start codon is boxed.

To confirm causality of the BCL11B p.R3S substitution for craniosynostosis and to generate a genetic model of the disorder, we introduced the c.7C>A mutation into the germ line of C57BL/6 mice using CRISPR-Cas9 (clustered, regularly interspaced, short palindromic repeat/CRISPR-associated protein 9) genome editing (40) as outlined in Figure 6 and described in the Reagents and Techniques section.

**Figure 7:**
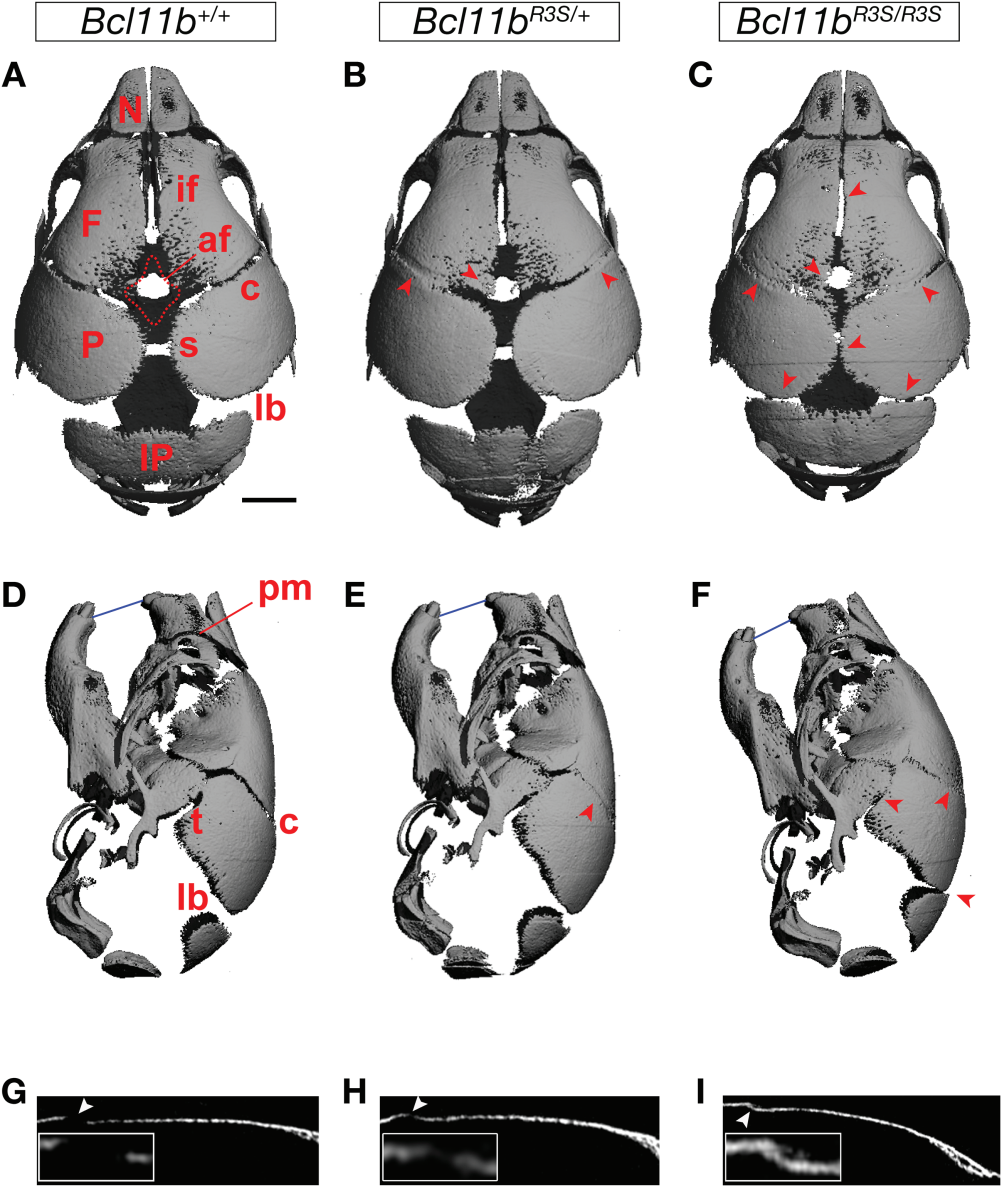
Craniosynostosis in mice harbouring p.R3S substitution. Superior (**A–C**), lateral (**D–F**) and sagittal sectional (**G–I**) views of micro-CT scans of wild-type control, *Bcl11b*^R3S/+^ and *Bcl11b*^R3S/R3S^ in P0 mice skulls. Arrowheads indicate increased mineralization and reduced anterior fontanel (af) and affected coronal (c), interfrontal (if), lambdoid (lb), premaxillary-maxillary (pm), sagittal (s) and temporal (t) sutures. Calvarial interparietal (IP), frontal (F), nasal (N) and parietal (P) bones are indicated in the figure, and the blue line indicates dental occlusion. Scale bar, 1 mm.

Heterozygous, *Bcl11b^R3S/+^* mice were born at Mendelian ratios, survived into adulthood without gross anatomical abnormalities and bred normally. However, examination of calvarial sutures by micro-CT revealed that *Bcl11b^R3S/+^* mice exhibited variable and partial, bilateral osteogenic fusion of the coronal suture that was accompanied by narrowing of the sagittal and lambdoid sutures by ~50% at P0 (compare panels A with B, D with E and G with H of Fig. 7; Supplementary Material, Figs S1 and S2). Other calvarial and facial sutures in the *Bcl11b^R3S/+^* mice were indistinguishable from those of wild-type mice (Fig. 7A–D; Supplementary Material, Fig. S3).

Homozygous, mutant mice (*Bcl11b^R3S/R3S^*) recapitulated perinatal lethality of *Bcl11b^−/−^* mice, due to apparent respiratory insufficiency (26), as well as multi-suture craniosynostosis at P0 involving the coronal (bilateral), interfrontal, sagittal, interparietal and temporal sutures (Fig. 7C, F and I). However, in marked contrast to *Bcl11b^−/−^* mice or those lacking BCL11B in cells derived from neural crest (29), *Bcl11b^R3S/R3S^* mice did not exhibit fusion of facial sutures (Fig. 7F; Supplementary Material, Fig. S3). The sutural mesenchyme of the facial skeleton is the most sensitive to loss of BCL11B and frequently undergoes premature osteogenic differentiation in *Bcl11b^+/−^* heterozygotes (29).

## Discussion

This paper describes a novel, *de novo* mutation of *BCL11B* in a patient with unilateral coronal suture craniosynostosis. The patient suffered severe sequelae requiring three corrective surgeries before the age of 13 years, suggesting a significant and progressive clinical course. The patient was negative for mutations of known craniosynostosis-related genes, which together with our previous demonstration that *Bcl11b^−/−^* mice exhibit craniosynostosis, including of the coronal suture (29), led us to evaluate the molecular consequences of the BCL11B p.R3S substitution and generate a mouse model for this mutation.

BCL11B contains an amino-terminal motif shared among a class of NuRD complex interacting transcription factors and the p.R3S substitution replaces Arg-3 of this highly conserved motif with serine (Fig. 1J). Crystal structures of RBBP4 in complex with the amino-terminal fragments of FOG-1 (34), BCL11A (36) and SALL4 (37) showed the motif residues Arg-3, Arg-4 and Lys-5 coordinated to the acidic core and surface of RBBP4 (Fig. 3A), suggesting that the Arg-3 contributes to the ionic coordination stabilizing its interaction with RBBP4 and closely related RBBP7. In these structures, Arg-3 appeared to be more flexible than the other motif invariant residues. Its electron density was not resolved in the ZNF827–RBBP4 structure (38), and unlike Arg-4 and Lys-5, the side-chain electron densities of Arg-3 fit to four slightly different coordination positions among the three resolved structures. Substitutions at Arg-3 are also more sensitive to context than other motif invariant residues. Site-directed mutagenesis of FOG-1 showed that Arg-4 and Lys-5 were critical for both high-affinity interaction with NuRD complex and transcriptional repressive activity. In contrast, mutation of Arg-3 selectively affected the affinity of FOG-1 for subtypes of NuRD complexes, contributing more to the stability of MTA2–NuRD than to MTA1–NuRD complexes (41). Moreover, mutation of Arg-3 only partly impaired FOG-1-mediated transcriptional repressive activity (41). However, Arg-3, Arg-4 and Lys-5 contribute equally to FOG-2-mediated transcriptional repression, suggesting that the individual residues of these motifs may exhibit a degree of contextual specificity (42). We found the p.R3S substitution in BCL11B eliminated detectable interaction with NuRD complex, also with the RBBP4/7-containing PRC2 complex, without affecting its interaction with SIRT1 (Fig. 2B and C), an interaction previously shown to involve an internal BCL11B domain independent of the amino terminus (33). BCL11B harbours multiple protein–protein interactions domains (33,43–46), and the present results suggest that the effect of the p.R3S substitution may be limited to a subset of interactions that depend on the amino-terminal motif of BCL11B with RBBP4/7-containing transcriptional regulatory complexes.

We examined the interaction of BCL11B and BCL11B p.R3S peptides with a subassembly of the NuRD complex composed of RBBP4 and a large fragment of MTA1. Our *in vitro* binding data demonstrated that the p.R3S peptide bound to the RBBP4–MTA1 with an affinity that was nearly an order of magnitude less than that of the corresponding wild-type peptide (Fig. 3B). Furthermore, results of MD simulations suggested that the p.R3S substitution additionally induced a conformational change in the BCL11B peptide, increased its disorder, negatively affected binding stability and potentially increased its potential for ectopic PTM (Fig. 5A and B).

Other interactions within the NuRD–BCL11B complex could positively or negatively impact interaction of wild-type BCL11B and/or BCL11B p.R3S proteins with the RBBP4–MTA1 complex. Based on previous structural studies of this subassembly (35) and a report that BCL11B interacts with MTA1 and MTA2 (45), it is conceivable that an MTA protein bound to RBBP4 could contribute to the stability of BCL11B interaction with RBBP4. Indeed, modelling results (Fig. 4) suggest that MTA1 region 466–468 may interact with Leu-12 and Ser-13 of BCL11B to stabilize the interaction of the latter with the holo-NuRD complex in the metazoan nucleus. Unfortunately, the available structural data does not include regions of MTA1/2 that could interact with both RBBP4/7 and the amino-terminal motif containing the substitution site.

**Figure 8:**
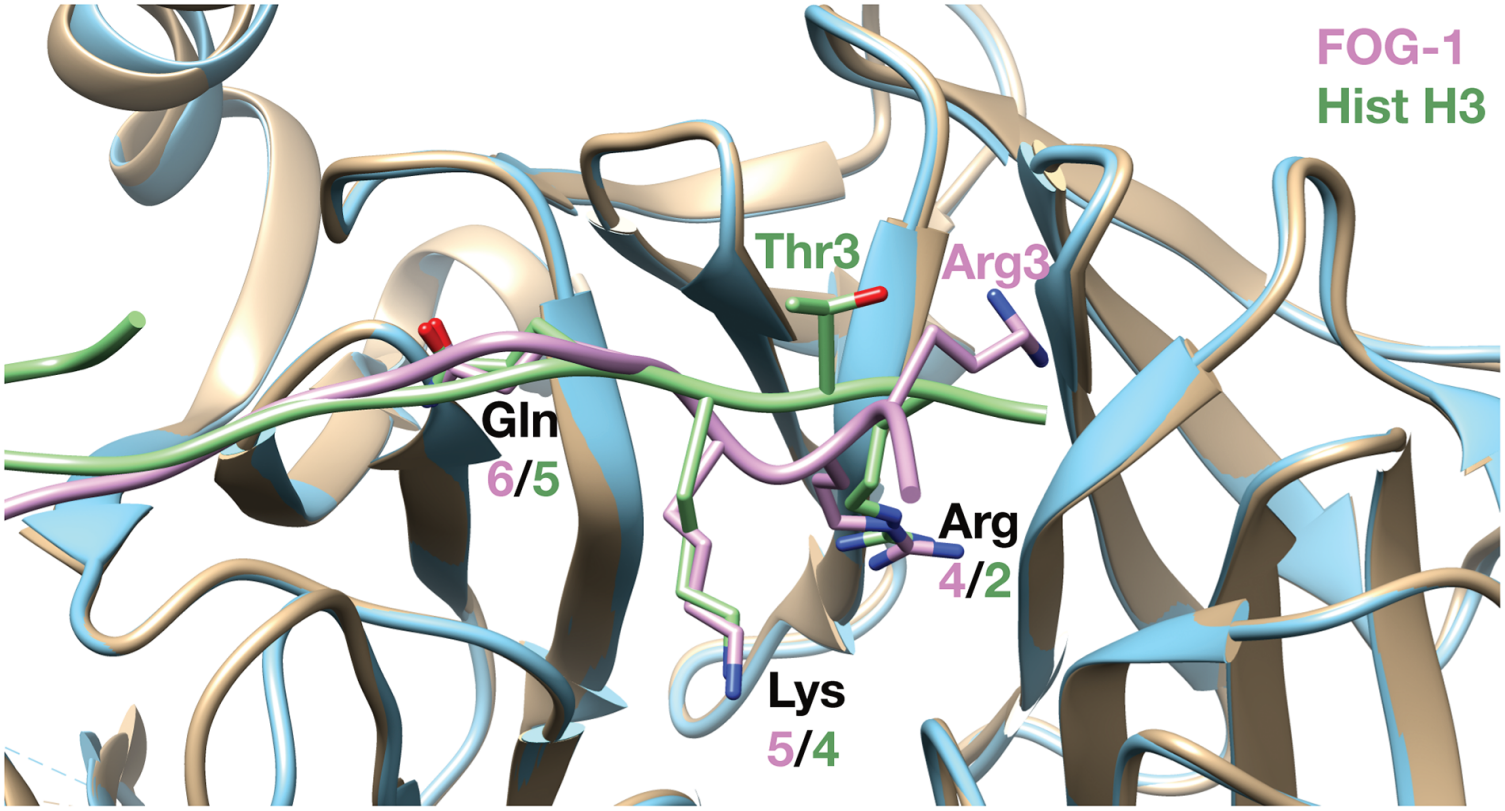
Common structural motif shared with histone H3 tail. The structural alignment of FOG-1 (pink) bound to RBBP4 (blue; PDB ID: 2XU7) (34) and histone H3 (green) bound to RBBP4/7 *Drosophila* homolog (tan; PDB ID: 2YBA) (71) is shown. BCL11B shares an identical sequence with FOG-1 (structure shown) through Gln-6 (see Fig. 1J).

The amino termini of BCL11B and related transcription factors share sequence (Fig. 1J) and structural (Fig. 8) homology with the amino-terminal tail of histone H3 when bound to RBBP4/7 factors. One consequence of the BCL11B p.R3S substitution is to expose both the mutant Ser-3 and the adjacent Ser-2 residues to solvent, similar to the position of Thr-3 histone H3 when bound to RBBP4/7 (Fig. 8). In histone H3, Arg-2 and Lys-4 are both methylated and Thr-3 is phosphorylated. With the exception of Ser-2 phosphorylation in transiently transfected cells (39), similar PTMs of the amino tail of BCL11B are unknown, but it is conceivable that the p.R3S substitution renders the amino tail of BCL11B susceptible to other, histone-like PTMs, such as ectopic phosphorylation of the mutant p.R3S site.

Disruption of the interaction of BCL11B with RBBP4 and RBBP7 could have profound effects on the transcriptional regulatory activity of BCL11B. BCL11B, a sequence-specific transcription factor, presumably recruits the RBBP4/7-containing complexes to specific genetic loci that are transcriptionally regulated by BCL11B. The reduced affinity of BCL11B p.R3S for RBBP4/7 could greatly compromise BCL11B-dependent recruitment of RBBP4/7-containing transcriptional regulatory complexes to chromatin, resulting in targeted dysregulation of gene expression. Moreover, the sequential nature of recruitment of RBBP4/7-containing transcriptional complexes to target chromatin may serve to amplify the dampened affinity of BCL11B p.R3S for RBBP4/7. For example, BCL11B-mediated repression of a subset of genes may involve sequential NuRD-mediated deacetylation of H3K27ac followed by PRC2-dependent methylation of H3K27, generating a transcriptionally non-permissive environment underpinned by the H3K27me3 modification (47–49). H3K27me3 locks down a transcriptionally repressive state that is physiologically critical in development, cellular differentiation and inactivation of the X chromosome (50–52). As BCL11B-mediated recruitment of both NuRD and PRC2 to this hypothetical locus presumably requires the sequential and binary interaction of BCL11B with RBBP4 (or RBBP7), the p.R3S substitution reported herein could have a multiplicative effect on the transcriptional outcome at this locus. This phenomenon may explain, at least partially, strong ectopic expression—or de-repression—of *Runx2* and/or *Fgfr2c* within the non-osteogenic mesenchyme of the coronal suture in *Bcl11b^−/−^* mice at E16.5, followed by ossification of this suture by E18.5 (29).

We generated a mouse model for the BCL11B p.R3S substitution, and this single amino acid change recapitulated coronal suture craniosynostosis in both the heterozygous and homozygous backgrounds. However, craniosynostosis in *Bcl11b^R3S/+^* mice was much less severe and more highly variable than that observed in homozygous *Bcl11b^R3S/R3S^* mice. These findings demonstrate that the BCL11B p.R3S substitution is causally related to the craniosynostosis seen in our patient.

Neither *Bcl11b^R3S/+^* nor *Bcl11b^R3S/R3S^* mice exhibited synostosis of sutures within the facial skeleton. This finding is surprising because we have previously found that facial sutures are even more sensitive to BCL11B dosage than those of the cranial skeleton (29), suggesting that the transcriptional regulatory complexes that BCL11B may work through to control suture patency in the facial skeleton are different from those required by the transcription factor to regulate cranial suture patency. Consistent with this speculation, our co-immunoprecipitation studies indicated that the p.R3S substitution selectively affected interaction of BCL11B with RBBP4- and RBBP7-containing complexes (NuRD and PRC2), but did not affect interaction with complexes that require other regions of BCL11B for interaction, e.g. the class III HDAC SIRT1 (33). Thus, the *Bcl11b^R3S/R3S^* mouse model generated herein may provide a means to dissect the mechanistic basis of BCL11B action in vivo and in several other developmental systems in which the transcription factor plays a critical role.

Recent reports (14,15) have described 11 monoallelic variants at the *BCL11B* locus that were linked to craniofacial dysmorphisms, neurodevelopmental pathologies, intellectual disabilities, dental anomalies and dermal defects. To date, only one of these, a p.N441K substitution in one of the zinc finger domains of BCL11B, has been investigated mechanistically and is reported to impact binding of BCL11B to known regulatory regions of the genome, alter the specificity of DNA binding by BCL11B and possibly generate a dominant-negative form of the protein (14). Unlike the patients who have multiple developmental anomalies, the patient with craniosynostosis described herein was otherwise healthy and did not present with any extra-craniofacial pathologies (14,15). In fact, the patient is now 19 years old and is continuing to undergo his formal education. Thus, the p.R3S substitution did not have a global effect on BCL11B function in this patient, suggesting that this mutation most likely affected the action of BCL11B primarily in the sutural mesenchyme. It will be of interest to examine the function of BCL11B p.R3S in other developmental systems, such as in the thymocyte, neuronal, skin and tooth lineages.

We hypothesize that the p.R3S substitution results in at least a partial or context-specific loss of BCL11B function, owing to restricted recruitment of NuRD and/or the RBB4/7-containing PRC2 complexes to BCL11B target loci, as described above. However, we cannot rule out a contribution to the phenotype through a gain of BCL11B function, in combination with the loss-of-function effects, in a promoter context-dependent manner. More broadly, mutations that eliminate any of the basic charges on Arg-3, Arg-4 or Lys-5, in BCL11B or in other amino-terminal motif-containing factors, are likely to disrupt interactions with RBBP4/7-containing NuRD and PRC2 complexes. There are two other reported variants affecting the charge on these residues beyond BCL11B p.R3S, namely SALL2 p.R3P and ZNF821 p.R4W (53), but the clinical significance of either substitution is unknown.

In conclusion, we identified a novel mutation in *BCL11B* associated with coronal suture synostosis, confirmed this association using a newly created mouse model and demonstrated that interaction of BCL11B with the transcriptional machinery was partially disrupted. This mouse model should be useful in dissecting the mechanistic basis for the role of BCL11B in maintenance of sutural patency, as well as in other organ systems in which BCL11B plays a critical developmental role.

## Materials and Methods

### Whole-genome sequencing

Ethical approval was given for WGS by the board of the Medical Ethical Committee Rotterdam (MEC-2012-140). Informed consent was received from the proband and parents.

WGS was performed on trio DNA by Complete Genomics (Mountain View, CA, USA), as described by Drmanac *et al.* (30). Data were analysed using CGA Tools version 1.6.0.43. A *de novo* disease model was tested, using the ‘calldiff script’ (Python script kindly provided by Complete Genomics) as described by Gilissen *et al.* (31). Prioritization of variants was based on the minimal somatic score and manual curation of the variants (Supplementary Material, Table S1). Confirmation of the *BCL11B de novo* variant was carried out by dideoxy sequencing. The *BCL11B* p.R3S variant has been deposited in the Leiden Open Variation Database (https://www.lovd.nl/BCL11B).

### Sequence analysis of *BCL11B* in craniosynostosis

The clinical study was approved by Oxfordshire Research Ethics Committee B (reference C02.143) and Riverside Research Ethics Committee (reference 09/H0706/20). Written informed consent to obtain samples for genetics research was obtained from each child’s parent or guardian. DNA was extracted from whole blood.

*BCL11B* was resequenced in 382 craniosynostosis probands negative for mutations in known craniosynostosis-related genes. Primer sequences, conditions used and bioinformatics analyses are included in Supplementary Material, Table S3. An additional 115 patients were screened for variants in the first exon of *BCL11B* by dideoxy sequencing (using standard protocols) or by targeted resequencing (using the primers for *BCL11B*-Ex1; Supplementary Material, Table S3).

### Reagents and techniques

#### Constructs

The BCL11B construct used herein has been described (54). BCL11B p.R3S was constructed from the parental plasmid using a mutagenic primer and the Q5 Site-Directed Mutagenesis Kit from New England Biolabs (NEB E0554S). The integrity of the entire open-reading frame of BCL11B was verified by dideoxy sequencing.

#### Generation of mutant mice

Mouse embryonic stem (ES) cells (E14Tg2aIV, 129/Ola) were transfected with pX458 (Addgene 48 138) (40) containing a guide sequence targeting a region near the *Bcl11b* translation start site (5′-CCGCAAACAGGGCAACCCGC-3′) and an oligonucleotide donor for homology directed repair and introduction of the c.7C>A mutation (5′- gcggcggcggcggctcagaccccctccccggcccgcatctgtgcagctttccgggcgatgccagaatagatgccgGggca**atg**tcc**A**g*ccgcaaacagggcaacccgc*agcacttgtcccagaggga-3′; uppercase G indicates site of introduction of a SacII restriction site for screening; the translation start site atg is in boldface; uppercase A denotes the mutation base). Briefly, ES cells were seeded onto gelatin-coated 6-well plates at 3 × 10^5^ cells per well 1 day before transfection with 3.3 μg plasmid, 10 μl of 10 μm donor complexed with 20 μl Fugene HD (Promega) oligonucleotide in a total volume of 100 μl made up with serum free medium. After 24 h, cells were sorted for green fluorescent protein-positive cells using fluorescence-activated cell sorting in a 96-well format. A homozygous c.7C>A ES clone was identified (containing a heterozygous SacII site), and karyotyping was injected into blastocysts (C57BL/6) for chimera generation. Several chimeric mice were produced, and the mutant allele was successfully transmitted through the germline (with and without the SacII site).

#### Specimen preparation and scanning

Skulls from P0 mice were collected and stored in 70% ethanol prior to scanning. Skulls were scanned using a Scanco μCT40 scanner (Scanco Medical AG, Basserdorf, Switzerland) at 12 μm voxel size, 55 kVp X-ray voltage, 145 μA intensity, with a 200 ms integration time as previously described (29). Stacks of images were also individually analysed in MicroView (version 2.5.0; GE Healthcare Biosciences, London, Ontario, Canada) to extract sagittal sections of the coronal and lambdoid sutures in all genotypes of mice. Mouse work was performed in accordance with UK Home Office regulations under approved project licences and the Oregon State University Institutional Animal Care and Use Committee.

#### Co-immunoprecipitations

HEK293T cells were transfected with vectors encoding FLAG-BCL11B (54), FLAG-BCL11B p.R3S or Myc-SIRT1 using the calcium phosphate method. Transfected cells were harvested in ice-cold phosphate-buffered saline (PBS) 48 h after transfection. Cell suspensions were centrifuged at 2000*g* for 5 min, followed by lysis under native conditions in buffer containing 20 mm HEPES, pH 7.4, 250 mm NaCl, 2 mm EDTA, 10% glycerol, 0.5% NP-40, 0.1 mm PMSF, 1 μg/mL pepstatin A, 5 μg/mL leupeptin, 10 μm E64, 25 mm NaF, 5 mm Na_2_P_2_O_4_ and 10 mm*N*-ethylmaleimide. Cells were incubated on ice for 30 min with vortexing every 5 min, followed by sonication on ice to complete lysis and shear DNA. The lysate was cleared by centrifugation, and BCL11B was immunoprecipitated from native extracts with anti-FLAG antibody (Sigma F4042), as previously described (54). SDS-PAGE immunoblot analyses used rat monoclonal anti-BCL11B (Abcam ab18465, 0.5 μg/ml) and the following rabbit primary antibodies: anti-MTA2 (Bethyl Laboratories A300-395A,1:5000 dilution), anti-HDAC2 (Bethyl A300-705A, 1:5000 dilution), anti-EZH2 (EMD-Millipore 07–689, 1:2000 dilution) and anti-Myc (Cell Signaling 2278, 1:1000 dilution to detect Myc-SIRT1), followed by IRDye680LT-conjugated anti-rat and IRDye800CW-conjugated anti-rabbit secondary antibodies (LI-COR Biosystems, Lincoln, NB, USA) used in combination. Fluorescent signal was recorded on an Odyssey dual-channel near-infrared imager (LI-COR) and analysed with manufacturer-supplied software (Image Studio, version 2.1).

#### Immunocytochemistry

HEK293T cells were grown on 0.1% gelatin-coated glass coverslips in 24-well plates seeded at 25 000 cells/well for 24 h prior to transfection with vectors encoding FLAG-BCL11B or FLAG-BCL11B p.R3S. Forty-eight hours after transfection, cells were washed twice with PBS, fixed in 4% paraformaldehyde in PBS for 10 min at room temperature, permeabilized with 0.3% Triton X-100/PBS for 10 min and washed twice with 0.1% Tween/PBS. The fixed and permeabilized cells were then prepared for immunocytochemistry by blocking for 1 h in 10% foetal bovine serum (FBS) in PBS, incubating for 4 h with rat anti-BCL11B monoclonal antibody (Abcam ab18465, 0.25 μg/ml) in 10% FBS/PBS, then incubating for 1.5 h with an anti-rat Cy3-conjugated secondary antibody (Jackson ImmunoResearch Laboratories, 1:500 dilution), followed by a final incubation in 10 μg/ml DAPI in PBS for 10 min. Cells were washed twice with 0.1% Tween/PBS between each immunocytochemistry step, and all incubations were conducted at room temperature. Cells were then washed twice with deionized water, followed by step-wise dehydration with 70% ethanol, 100% ethanol and xylene. Coverslips were then mounted on glass slides with DPX (VWR), dried overnight and stored at 4°C in the dark. Fluoromicrographs were obtained using a Zeiss microscope with appropriate filters and a 63× oil-immersion objective. Colour images were digitally overlaid using Zeiss AxioVs40 software, version 4.8.2.0.

#### Fluorescence anisotropy

Steady-state fluorescence anisotropy of fluorescein-labelled wild-type BCL11B and BCL11B p.R3S amino-terminal peptides and unlabelled RBBP4–MTA1 complex were recorded in black 96-well plates (Corning) at room temperature using a Victor X5 plate reader (Perkin Elmer). Synthetic BCL11B peptides (residues 1–15) were carboxy-terminal labelled with 5-carboxyfluorescein (5-FAM) and were purchased from Cambridge Research Biochemicals (Billingham, UK), and the complex of full-length RBBP4 and residues 464–546 of MTA1 were prepared as previously described (35). Measurements were conducted in buffer containing 50 mm Tris, pH 7.5, 150 mm NaCl, 0.15 mg/mL BSA and 0.03% Tween-20. Fluorescence was excited at 480 nm and monitored at 535 nm. For each concentration of RBBP4–MTA1, anisotropy, *A,* was calculated according to

[Eq. 1] \begin{equation*} A=\frac{I_{\Vert }-{I}_{\perp }}{I_{\Vert }+2{I}_{\perp }}, \end{equation*}

where *I*_‖_ is the fluorescence intensity measured in the parallel plane and *I*_⊥_ is the fluorescence intensity measured in the perpendicular plane. Anisotropy measurements were fit to the following equation:

[Eq. 2] \begin{equation*} A=\frac{b-\sqrt{b^2-4\left[L\right]{A}_{max}}}{2}, \end{equation*}

where [L] is the total concentration of RBBP4–MTA1 complex, *A_max_* is the anisotropy of the complex bound fluorescein-labelled peptide, *K* is the dissociation constant of the peptide and *b* = *A_max_* + *K* + [L].

#### Molecular modelling

MD simulations of the conserved amino-terminal tails of wild-type BCL11B and BCL11B p.R3S bound to the RBBP4–MTA1 complex were based on the high-resolution structures of FOG-1 peptide bound to RBBP4 (PDB ID: 2XU7) (34) and the RBBP4–MTA1 complex using PDB IDs 4PBY (55) and 5FXY (35). Modeller version 9.17 (56,57) and SCWRL4 (58) were used to generate initial simulation models consisting of residues 1–15 of BCL11B and BCL11B p.R3S, 8–411 of RBBP4 and 464–546 of MTA1. MD simulations used the GROMACS software package, version 2016 (59) and were conducted in explicit solvent of 0.15 m NaCl in TIP3P model of water, neutralized with additional sodium ions and parameterized with the CHARMM36m all-atom force field (60), which was chosen to model the inherently disordered BCL11B peptides. The LINCS algorithm (61) was used to constrain all bonds; short-range Coulombic and van der Waals non-bonded interactions were cut off at 1.2 nm, and long-range electrostatics were calculated according to the particle-mesh Ewald algorithm (62). Periodic boundary conditions were applied to all dimensions.

The modelled peptide–complex systems were equilibrated to simulation conditions following steepest descent energy minimization in two steps with positional restraints placed on the protein heavy atoms. First, the temperature was stabilized under a canonical constant volume (NVT) ensemble for 100 ps. Subsequently, pressure was stabilized under isothermal–isobaric ensemble (NPT) for 200 ps. Protein and solvent were coupled to separate temperature baths and maintained with the Nosé–Hoover thermostat (63,64) at 310 K, and pressure was maintained at 1 bar with the Parrinello–Rahman barostat (65). Following equilibration, unrestrained MD simulations were conducted for 100 ns to generate initial configurations for steered molecular dynamics (SMD) simulations (66). During each SMD simulation, the RBBP4 centre of mass was fixed and BCL11B peptides were pulled along the axis orthogonal to the peptide binding site by a force attached to the peptide centre of mass with a harmonic spring constant of 1000 kJ/mol/nm^2^ at a rate of 0.001 nm/ps for 3.5 ns. Starting configurations for umbrella sampling equilibrium MD were extracted from these SMD trajectories to provide a distribution of overlapping sampling windows along the SMD reaction coordinate. Each extracted configuration was then re-equilibrated with positional restraints placed on protein-heavy atoms under NPT ensemble for 100 ps prior to 15 ns of unrestrained MD simulation. The weighted histogram analysis method (67,68) was used to calculate the one-dimensional PMF as a function of centre-of-mass separation.

## Supplementary Material

Suppl_FigsTables_Goos-etal_2019-01-28_ddz072
