# Supplementary material for: A de novo substitution in BCL11B leads to loss of interaction with transcriptional complexes and craniosynostosis
Source: Hum Mol Genet. 2019 Apr 3;28(15):2501–13. doi: 10.1093/hmg/ddz072 (PMC6644156; doi:10.1093/hmg/ddz072)
Supplement: Suppl_FigsTables_Goos-etal_2019-01-28_ddz072 [file suppl_figstables_goos-etal_2019-01-28_ddz072.pdf]

Supplementary Material

A *de novo* point mutation in BCL11B leads to loss of interaction with transcriptional complexes and craniosynostosis

Jacqueline A.C. Goos, Walter K. Vogel, Hana Mlcochova, Christopher J. Millard, Elahe Esfandiari, Wisam Hussein Selman, Eduardo Calpena, Nils Koelling, Evan L. Carpenter, Sigrid M.A. Swagemakers, Peter J. van der Spek, Theresa M. Filtz, John W.R. Schwabe, Urszula T. Iwaniec, Irene M.J. Mathijssen, Mark Leid, and Stephen R.F. Twigg

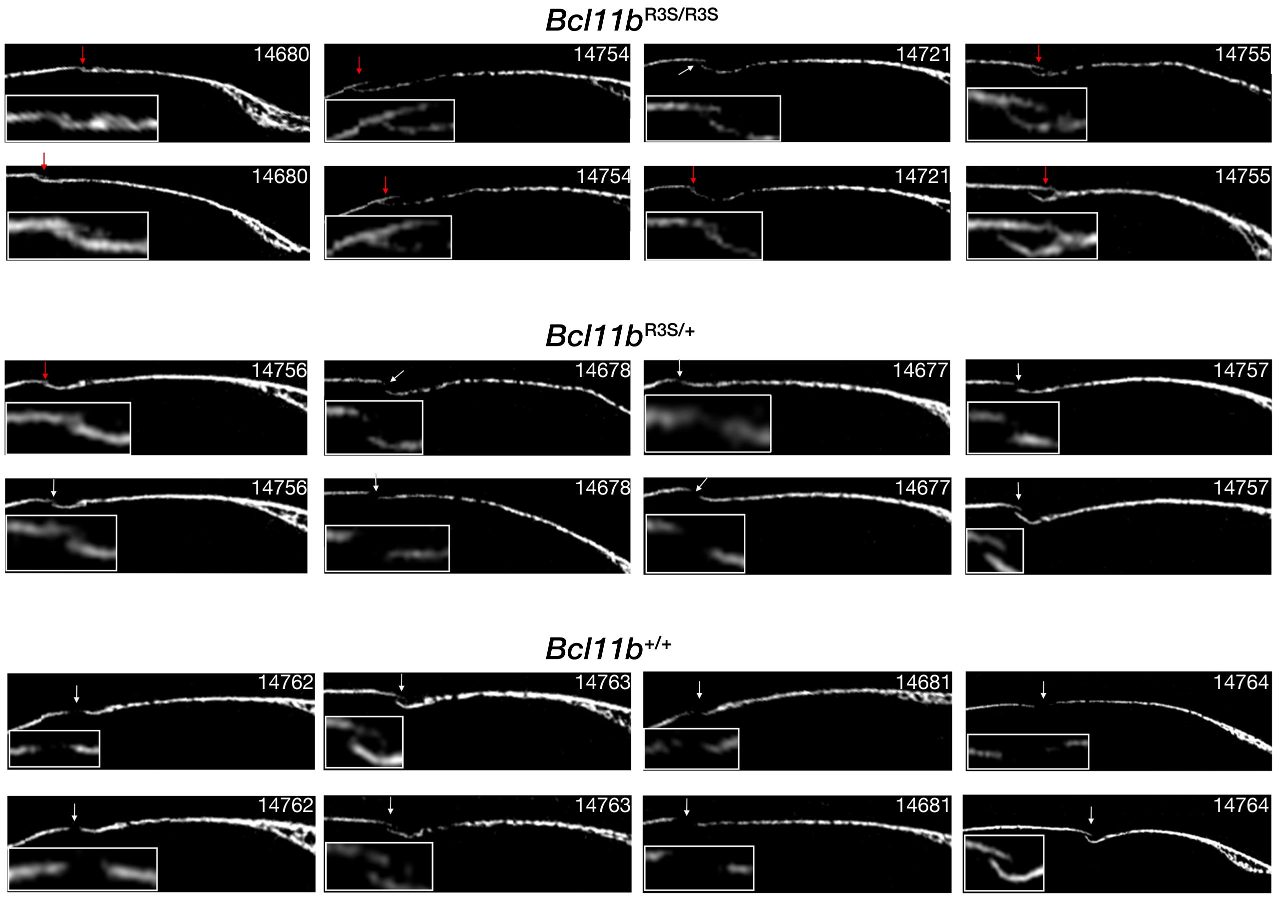

Figure S1. Micro-CT analysis of mice skulls harbouring *Bcl11b* p.R3S substitution. The analysis shows representative sagittal sections from four mice of indicated genotype. The location of coronal suture is indicated with an arrow and shown at higher magnification inset into each panel. Fusions of the coronal suture are indicated with a red arrow and the number in each panel identifies the individual mouse shown.

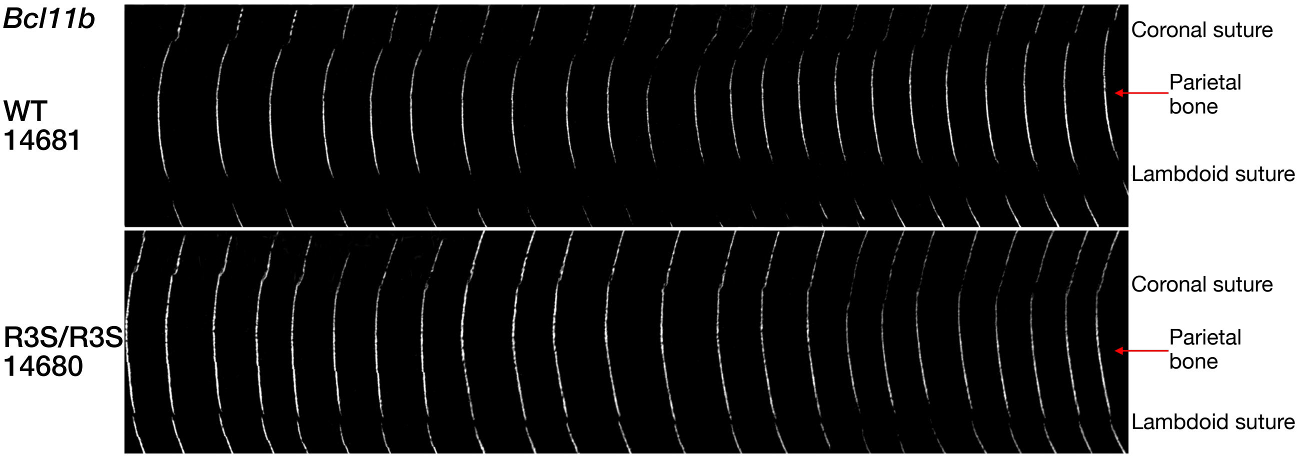

Figure S2. Micro-CT analysis of serial sagittal sections of coronal and lambdoid sutures. Comparative analysis of wild-type and *Bcl11b*$^{R3S/R3S}$ mice showing fusion of the coronal suture and narrowing of the lambdoid sutures in the latter.

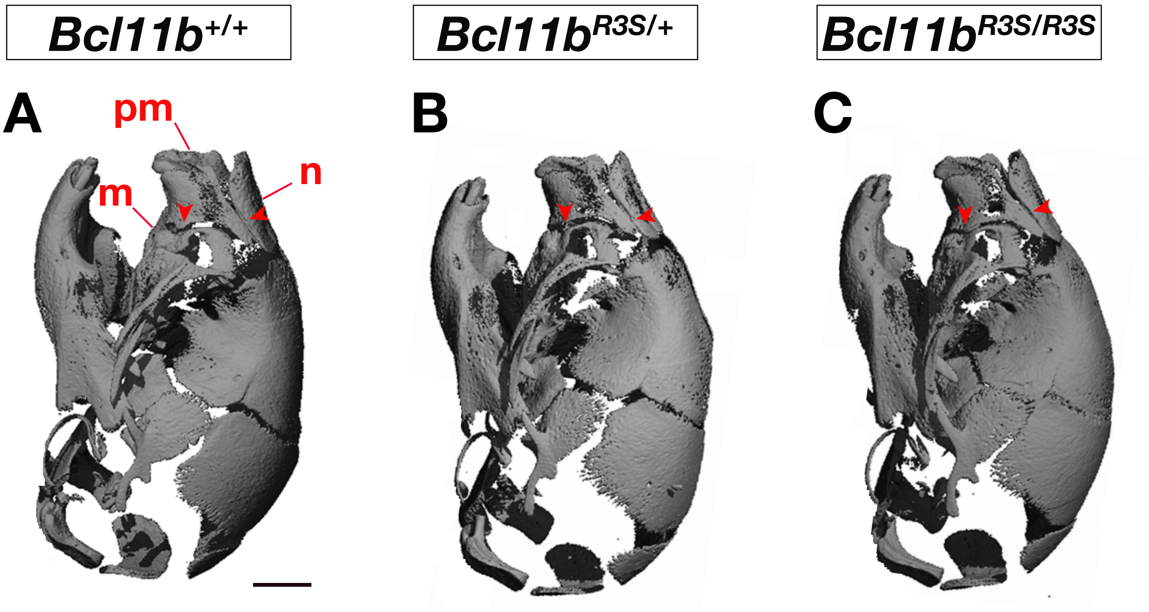

Figure S3. Facial sutures of mouse skulls harbouring *Bcl11b* p.R3S substitution. Micro-CT images at P0 revealed lack of fusion of facial sutures in both *Bcl11b*$^{R3S/+}$ and *Bcl11b*$^{R3S/R3S}$ mice, a prominent feature of *Bcl11b*$^{+/-}$ and *Bcl11b*$^{-/-}$ mice (1). Images were tilted to allow clear views of the facial skeleton. Arrows denote naso-premaxillary and premaxillary-maxillary sutures. Abbreviations: premaxillary (pm), maxillary (m), and nasal (n) bones. Scale bar = 1 mm.

**Table S1. High confidence coding *de novo* variants from WGS[a]**									
gene	Variant type[b]	chr	CRCh37/hg19 begin	CRCh37/hg19 end	reference allele	allele 1	allele 2	minimal Somatic Score	CADDScore [c]
BCL11B	SNP	chr14	99737548	99737549	G	T (p.R3S)	G	18	24.2
FERMT2[d]	SNP	chr14	53348144	53348145	G	C (p.T190S)	G	10	14.9

Note—[a]Data were analysed using *cga tools*, version 1.6.0.43. A *de novo* disease model was hypothesized, using the '*calldiff* script' (Python script kindly provided by Complete Genomics) as described by Gilissen *et al.* (2)—this algorithm identified two high confidence *de novo* variants in *BCL11B* and *FERMT2*. [b]SNP = Single Nucleotide Polymorphosm. [c]CADD scores were described by Kircher *et al.* (3). [d]This *FERMT2* variant was considered unlikely to be the major contributor to the phenotype for the following reasons: *FERMT2* is not a known disease gene, the T190S change is conservative and substitution of this residue to asparagine (p.T190N) has been observed four times in control databases (gnomAD; 4/276830 alleles). By contrast, BCL11B is expressed in cranial sutures, deletion in mice leads to craniosynostosis, it is a known disease gene, and the variant, which has a much higher CADD score, is within a highly conserved functional domain. However, FERMT2 has been shown to have a role in chondrogenesis and bone formation (4), so we cannot exclude some contribution to the phenotype.

**Table S2. *BCL11B* variants identified by resequencing**													
Proband	Cranio-synostosis	Sutures	Variant[a]	cDNA, Protein	Del. Score[b]	Polyphen2	SIFT	Located in functional domain?	ExAC[c] frequency	ExAC alleles	gnomAD[d] frequency	gnomAD alleles	Inheritance?
1274	NSMultiple	Not specified	chr14: 99641690C>T	c.1483G>A p.A495T	2	Benign	Tolerated	No	1.6E–5	40 / 24410	5.01E–4	100 / 199614	not assayed
2179	SMultiple	Not specified	chr14: 99723843G>A	c.392C>T p.T131M	5	Possibly damaging	Damaging (Low confidence)	No	5.09E–5	6 / 117806	3.26E–05	9 / 276194	not assayed
6959	SS	Sagittal	chr14: 99724153C>T	c.82G>A p.A28T	1	Benign	Tolerated	No	1.80E–5	2 / 110828	3.23E–05	1 / 30962	not assayed
6262	SMultiple	Sagittal, RC	chr14: 99642335T>C	c.838A>G p.M280V	2	Benign	Tolerated	No	Novel	Novel	Novel	Novel	From unaffected father

Note—[a]GRCh37/hg19. [b]Del = deleterious; the severity of amino acid substitutions was predicted using a combination of the following 6 metrics: Polyphen2, SIFT, PhyloP, LRT, Mutation Taster, and GERP++ (5). [c]ExAC, variant frequency from the Exome Aggregation Consortium (http://exac.broadinstitute.org). [d]gnomAD – Genome Aggregation Database variant frequency (http://gnomad.broadinstitute.org).

**Table S3. Targeted resequencing of *BCL11B* in craniosynostosis[a]**				
Amplicon	Primer sequence 5′→3′		Length excluding adapters	Multiplexed PCR mix
	Forward	Reverse	(bp)	
BCL11B Ex 1	ACACTGACGACATGGTTCTACACCATCAGTGCAGCTCTCCG	TACGGTAGCAGAGACTTGGTCTGTAGACTCTGCCAGCCAGC	233	Mix A
BCL11B Ex2	ACACTGACGACATGGTTCTACAAAGTTGGGTGCTGTGACCG	TACGGTAGCAGAGACTTGGTCTACAGCAACCAGGCAAGCG	473	Mix C
BCL11B Ex3	ACACTGACGACATGGTTCTACAGCCTGTGTCCCGATTGCC	TACGGTAGCAGAGACTTGGTCTACCTTGCTCCAGCGCTGC	453	Mix B
BCL11B Ex4.1	ACACTGACGACATGGTTCTACAACAGACCTGCGTCTTCGG	TACGGTAGCAGAGACTTGGTCTTGCGCAGCAGGTTGAAGG	442	Mix C
BCL11B Ex4.2	ACACTGACGACATGGTTCTACATGAATTTCCTGGGCGACAGC	TACGGTAGCAGAGACTTGGTCTCTGGAACTTGAAGGTCTTGCC	479	Mix A
BCL11B Ex4.3	ACACTGACGACATGGTTCTACACAAGTCGTGCGAGTTCTGC	TACGGTAGCAGAGACTTGGTCTTCCGAGTCCATGCTGAAGC	405	Mix C
BCL11B Ex4.4	ACACTGACGACATGGTTCTACAAGCTGCTACTGGAGAACGAG	TACGGTAGCAGAGACTTGGTCTTCTCGGACGGGATGAGCG	489	Mix B
BCL11B Ex4.5	ACACTGACGACATGGTTCTACAGAGAAGGACCTGGAGCTGC	TACGGTAGCAGAGACTTGGTCTACAGCTCGCACTTGTAAGGC	415	Mix A
BCL11B Ex4.6	ACACTGACGACATGGTTCTACACGACACGTGCGAGTACTGC	TACGGTAGCAGAGACTTGGTCTGGAGGCAAGTCAGGTCAGC	378	Mix B

Note—[a]Primer pairs were designed to amplify the coding regions of the four exons and the intron/exon boundaries of *BCL11B* (NM_138576.3, ENST00000357195). Universal CS1 (ACACTGACGACATGGTTCTACA) and CS2 (TACGGTAGCAGAGACTTGGTCT) adaptor sequences were included at the 5′ ends of all target-specific forward and reverse primers, respectively. To reduce the amount of genomic DNA used, target regions were amplified using multiplexed PCR. PCR products were amplified from 20 ng of genomic DNA using FastStart Taq DNA Polymerase (Roche). For each patient, the multiplexed PCR products were combined, diluted 100-fold and used in a second PCR reaction to incorporate Illumina sequence-specific adaptors and sample indexes (100-4876 Access Array, Fluidigm), for 9 cycles using the Q5 High-Fidelity DNA Polymerase (NEB). The indexed PCR products were pooled, purified with AMPure XP beads (Beckman Coulter Ltd.) and quantified using an Agilent 2200 TapeStation (Agilent Technologies) with High Sensitivity D1000 ScreenTape and Qubit dsDNA HS Assay Kit (Qubit). Pooled libraries were diluted to a final concentration of 9 pM and sequenced using MiSeq 500 cycle Reagent Kits (with 2x255 paired-end reads) on the MiSeq platform (Illumina, CA, USA). The following custom primers were used for sequencing on the MiSeq: custom CS1-Seq primer (0.5 µM) for read 1 (5′- A+CA+CTG+ACGACATGGTTCTACA -3′, where + indicates a LNA (Locked Nucleic Acid)-modified nucleotide, CS2-Seq primer for read 2 (5′- T+AC+GGT+AGCAGAGACTTGGTCT -3′) and RC-CS2 primer for the indexing read (5′- A+GAC+CA+AGTCTCTGCTACCGTA -3′). The raw sequencing reads were aligned to the GRCh37/hg19 reference genome. Variant calls and coverage information were obtained using a custom pipeline: Reads were trimmed to remove primers and low-quality basecalls and aligned to GRCh37 (without alt contigs) using *bwa mem,* version 0.7.12 (6, 7). Coverage was calculated using *BEDtools,* version 2.25.0 (8), requiring a minimum coverage of 10 to consider a target region fully covered. Variants were called with *Platypus,* version 0.8.1 (9) and annotated using *Annovar,* version 2015-06-17 (10). Further processing and annotation was performed using custom scripts written in Python 3.5.3 with *pysam* (http://pysam.readthedocs.io/en/latest/index.html), *biopython* (11), and *pandas* (http://pandas.pydata.org/index.html). The entire pipeline, built using *Snakemake,* version 3.11.2 (12), is available upon request.

References

1. Kyrylkova, K., Iwaniec, U.T., Philbrick, K.A. and Leid, M. (2016) BCL11B regulates sutural patency in the mouse craniofacial skeleton. *Dev. Biol.*, **415**, 251–260.

2. Gilissen, C., Hehir-Kwa, J.Y., Thung, D.T., van de Vorst, M., van Bon, B.W.M., Willemsen, M.H., Kwint, M., Janssen, I.M., Hoischen, A., Schenck, A., *et al.* (2014) Genome sequencing identifies major causes of severe intellectual disability. *Nature*, **511**, 344–347.

3. Kircher, M., Witten, D.M., Jain, P., O'Roak, B.J., Cooper, G.M. and Shendure, J. (2014) A general framework for estimating the relative pathogenicity of human genetic variants. *Nat. Genet.*, **46**, 310–315.

4. Wu, C., Jiao, H., Lai, Y., Zheng, W., Chen, K., Qu, H., Deng, W., Song, P., Zhu, K., Cao, H., *et al.* (2015) Kindlin-2 controls TGF-β signalling and Sox9 expression to regulate chondrogenesis. *Nat Commun*, **6**, 7531.

5. Fu, W., O'Connor, T.D., Jun, G., Kang, H.M., Abecasis, G., Leal, S.M., Gabriel, S., Rieder, M.J., Altshuler, D., Shendure, J., *et al.* (2013) Analysis of 6,515 exomes reveals the recent origin of most human protein-coding variants. *Nature*, **493**, 216–220.

6. Li, H. and Durbin, R. (2009) Fast and accurate short read alignment with Burrows-Wheeler transform. *Bioinformatics*, **25**, 1754–1760.

7. Li, H. (2013) Aligning sequence reads, clone sequences and assembly contigs with BWA-MEM. *arXiv*, **q-bio.GN**, 1303.3997v2.

8. Quinlan, A.R. (2014) BEDTools: The Swiss-Army Tool for Genome Feature Analysis. *Curr. Protoc. Bioinformatics*, **47**, 11.12.1–11.12.34.

9. Rimmer, A., Phan, H., Mathieson, I., Iqbal, Z., Twigg, S.R.F., WGS500 Consortium, Wilkie, A.O.M., McVean, G. and Lunter, G. (2014) Integrating mapping-, assembly- and haplotype-based approaches for calling variants in clinical sequencing applications. *Nat. Genet.*, **46**, 912–918.

10. Wang, K., Li, M. and Hakonarson, H. (2010) ANNOVAR: functional annotation of genetic variants from high-throughput sequencing data. *Nucl. Acids Res.*, **38**, e164.

11. Cock, P.J.A., Antao, T., Chang, J.T., Chapman, B.A., Cox, C.J., Dalke, A., Friedberg, I., Hamelryck, T., Kauff, F., Wilczynski, B., *et al.* (2009) Biopython: freely available Python tools for computational molecular biology and bioinformatics. *Bioinformatics*, **25**, 1422–1423.

12. Köster, J. and Rahmann, S. (2012) Snakemake—a scalable bioinformatics workflow engine. *Bioinformatics*, **28**, 2520–2522.
